# funbarRF: DNA barcode-based fungal species prediction using multiclass Random Forest supervised learning model

**DOI:** 10.1186/s12863-018-0710-z

**Published:** 2019-01-07

**Authors:** Prabina Kumar Meher, Tanmaya Kumar Sahu, Shachi Gahoi, Ruchi Tomar, Atmakuri Ramakrishna Rao

**Affiliations:** 1 Division of Statistical Genetics, ICAR-Indian Agricultural Statistics Research Institute, New Delhi, 110012 India; 2 Centre for Agricultural Bioinformatics, ICAR-Indian Agricultural Statistics Research Institute, New Delhi, 110012 India; 3 Department of Bioinformatics, Janta Vedic College, Baraut, Baghpat, Uttar Pradesh 250611 India

**Keywords:** BOLD systems, DNA barcode, ITS, Fungal taxonomy, CBOL

## Abstract

**Background:**

Identification of unknown fungal species aids to the conservation of fungal diversity. As many fungal species cannot be cultured, morphological identification of those species is almost impossible. But, DNA barcoding technique can be employed for identification of such species. For fungal taxonomy prediction, the ITS (internal transcribed spacer) region of rDNA (ribosomal DNA) is used as barcode. Though the computational prediction of fungal species has become feasible with the availability of huge volume of barcode sequences in public domain, prediction of fungal species is challenging due to high degree of variability among ITS regions within species.

**Results:**

A Random Forest (RF)-based predictor was built for identification of unknown fungal species. The reference and query sequences were mapped onto numeric features based on gapped base pair compositions, and then used as training and test sets respectively for prediction of fungal species using RF. More than 85% accuracy was found when 4 sequences per species in the reference set were utilized; whereas it was seen to be stabilized at ~88% if ≥7 sequence per species in the reference set were used for training of the model. The proposed model achieved comparable accuracy, while evaluated against existing methods through cross-validation procedure. The proposed model also outperformed several existing models used for identification of different species other than fungi.

**Conclusions:**

An online prediction server “funbarRF” is established at http://cabgrid.res.in:8080/funbarrf/ for fungal species identification. Besides, an R-package *funbarRF* (https://cran.r-project.org/web/packages/funbarRF/) is also available for prediction using high throughput sequence data. The effort put in this work will certainly supplement the future endeavors in the direction of fungal taxonomy assignments based on DNA barcode.

## Background

**Fig. 1:**
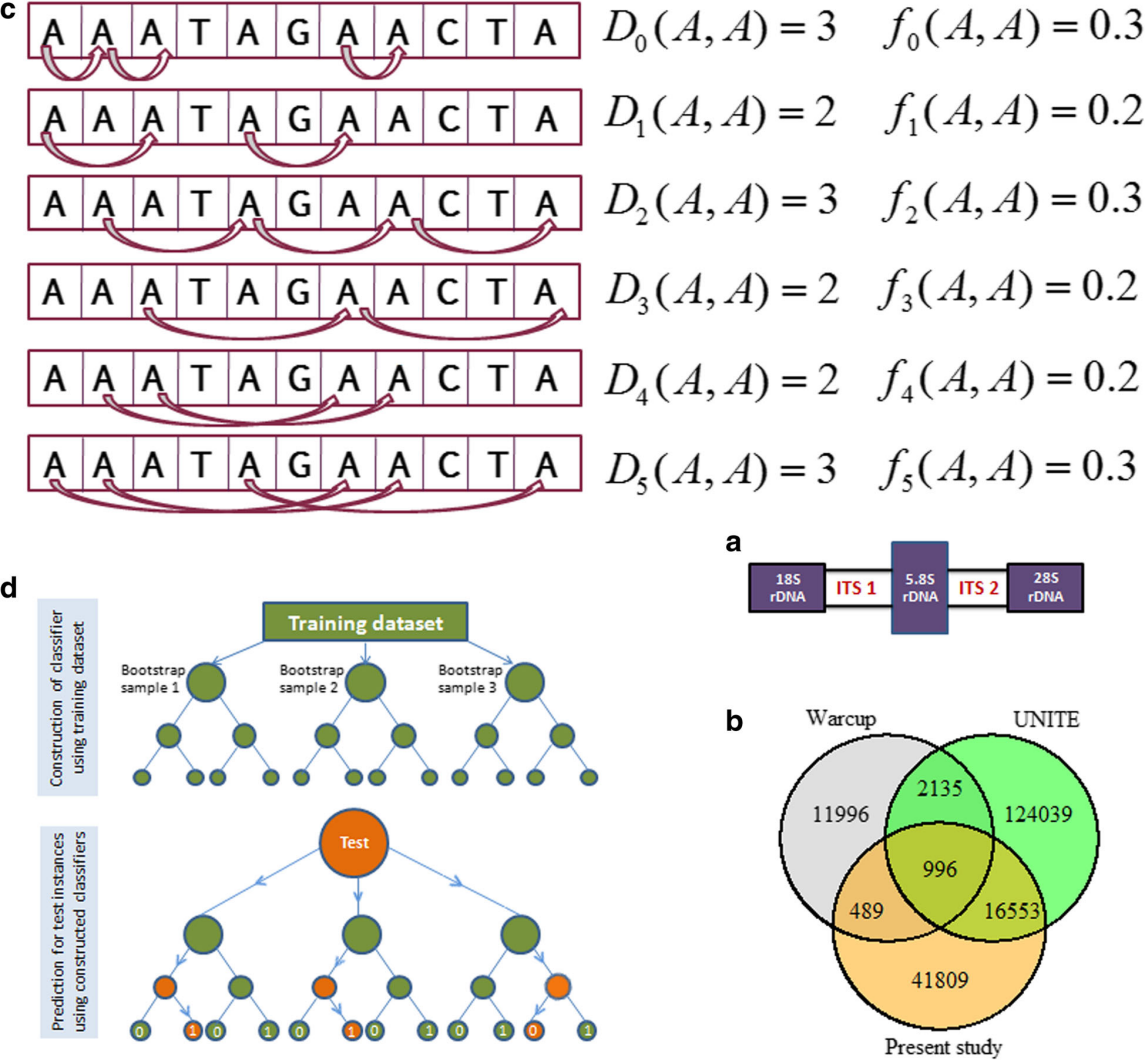
(**a**) Diagrammatic representation of ITS region of rDNA that includes ITS1 and ITS2 separated by 5.8s gene. (**b**) Venn diagram showing the number of sequences of this work present in other databases. **c** Diagrammatic representation of the computation of gapped base pair features of di-nucleotide AA. **d** Flow diagram showing the steps of training and testing involved in prediction using RF classifier. During training, tree-based classifiers are constructed on bootstrap samples of the training dataset, whereas in testing the test instance is dropped in every constructed classifier for predicting its label based on majority voting scheme

In meta-genomic studies, taxonomy classification is crucial for characterizing microbial communities [1]. In particular, prediction of unknown fungal specimens and conservation of their genomic resources are vital for studying and preserving fungal diversity [2]. However, identification of specimens that lacked morphological character is often difficult [3]. In this direction, molecular technique like DNA barcoding [4] has been successfully employed in the recent years for species identification [5–7]. In this technique, a standard genomic region is used to distinguish species based on barcode-gap [8]. The COI (cytochrome c oxidase subunit I) gene of mitochondrial DNA was first accepted as the barcode by the CBOL (consortium for barcode of life) [9] for prediction of animal species [3]. Later on, the *matK* and *rbcL* genes of chloroplast region were adopted by CBOL as barcodes for identification of plant species [10]. As far as fungus is concerned, the ITS of rDNA that includes ITS1 and ITS2 separated by 5.8S genic region (Fig. 1a), has been accepted by almost all the mycologists as the molecular region for species identification [11–13].

Considering the importance of barcoding in the preservation of species diversity as well as for other applications, the CBOL has been continuously emphasizing on the development of new approach(s) for identification of unknown species based on its barcode sequence [14, 15]. However, reference datasets having barcode sequences with known species labels are essential for the prediction of unknown species. For fungal species identification, two important ITS reference databases namely UNITE [13] and Warcup [16] have been developed. Besides, the BOLD (barcode of life data) [9] system also provides taxonomic information for fungal species identification. As far as prediction of fungal species is concerned, few computational approaches namely RDP classifier [16, 17], SINTAX [1], Mycofier [18] and MOTHUR [19] were proposed in the past. The RDP classifier employed naïve Bayes algorithm for taxonomy assignment, based on *k*-mer (*k*=8) similarity features [17]. Similar *k*-mer (*k*=8) features were also utilized in the SINTAX algorithm for taxonomy prediction by using a non-Bayesian classifier [1]. The *k*-nearest neighbor (*k*NN) algorithm was implemented in MOTHUR for taxonomy classification, based on *k*-mer (*k*=8) similarity measures [19]. In Mycofier, naïve Bayes classifier coupled with *k*-mer (*k*=5) features was adopted for identification of fungi at genus label [18].

Though concerted efforts have been put for the development of above mentioned tools and techniques that have advanced our knowledge for species identification using DNA barcode, still there is a room for further improvement. The 8-mer similarities have been adopted in RDP classifier, SINTAX and MOTHUR, where the number of features are large (i.e., 4^8^). So, prediction with same accuracy using less number of features is one of the aims of this study. Further, tool like PROTAX [7] depends upon the output of third party software BLAST [20], which itself takes longer time for performing sequence alignment for larger size dataset. Thus, the other aim of this work is to develop an alignment free tool for prediction of fungal species. Furthermore, the supervised machine learning techniques such as naïve Bayes classifier, kNN, Bayesisn regression model have been successfully employed for taxonomy assignments of fungal species, as evidenced from the above mentioned studies. Keeping above in mind, we have proposed a supervised learning-based prediction model for identification of fungal species, by analyzing their barcode sequences. In the proposed model, gapped base-pair compositions [21] were used as features and Random Forest (RF) [22] methodology as predictor. The performance of the developed model was not only evaluated with fungal species but also for the prediction of other species as well. We believe that the developed approach will supplement the existing tools and techniques for species identification using DNA barcode.

## Methods

### Barcode sequences of fungal species

The Warcup dataset (17878 sequences belonging to 8551 species) was used to test the predictive ability of the RDP classifier [16] and SINTAX algorithm [1]. Besides, the RDP classifier was also evaluated with UNITE dataset (145019 sequences belonging to 10297 species). Further, performance of another machine learning-based classifier i.e., Mycofier [18] was tested on fungal ITS sequences from the NCBI GenBank (https://www.ncbi.nlm.nih.gov/). None of the above studies have used fungal barcode sequences of BOLD systems (http://www.boldsystems.org/), which is one of the most wide spread endeavor in the field of barcode-based species identification [23]. Therefore, we preferred the BOLD database for collecting the fungal ITS sequences for our study. At first, 68565 barcode sequences belonging to 4182 species (at least 3 sequences per species), across all the 7 phyla of fungal kingdom were collected. Excluding sequences with non-standard nucleotide bases, 60348 sequences confined to 4100 species were obtained. Further excluding 330 species with 1 or 2 sequences, 3770 species with 59847 barcode sequences were retained for the analysis. Among 59847 sequences, more than 56000 are from ITS regions and rests are from other genomic portions (Table 1). Out of 59847 sequences, 1485 (2.481% of 59847) and 17549 (29.32% of 59847) sequences are found common with the Warcup and UNITE datasets respectively (Fig. 1b). So, the prepared dataset consists of ~70% non-redundant (with Warcup and UNITE) sequences (excluding the 18038 common sequences present in Warcup and UNITE datasets, which is 30.14% of 59847).

**Table 1:** Distribution of collected fungal barcode sequences over different genomic regions. It can be seen that >56000 sequences out of 59847 sequences are from ITS (including ITS1 and ITS2) region. These 59847 barcode sequences are belonged to 3770 species, where at least 3 sequences are present for each species.

Genomic region	18S	28S	5.8S	AOX-fmt	atp6	COI-5P	COII	COXIII	ITS	ITS1	ITS2
# Sequences	5	6	2418	79	3	595	3	3	51886	2428	2421

### Feature generation

Feature generation is a crucial step in computational predictions using biological sequences [24]. Since the biological sequences are the strings of alphabets, they should be transformed to numeric vectors before being employed as input in supervised learning-based predictors [25]. As far as barcode-based species identification using machine learning predictors is concerned, sparse encoding technique was adopted by Weitschek et al. [15]. In another study, Meher et al. [26] encoded the barcode sequences based on the composition of contiguous *k*-mer, for species identification using RF [22] machine learning technique. Specific to fungal species, *k*-mer features [26] were employed in RDP classfier, SINTAX algorithm and Mycofier for encoding barcode sequences into numeric vectors. Recently, Brinda et al. [27] shown that the spaced *k*-mer [21] provides significantly higher accuracy as compared to the contiguous *k*-mer. Therefore, in the present study, the *g*-spaced base pair features [21] were used to encode the barcode sequences into numeric feature vectors. Five kinds of *g*-spaced features namely 1-spaced (*g*=1), 2-spaced (*g*=2), 3-spaced (*g*=3), 4-spaced (*g*=4) and 5-spaced (*g*=5) were computed. This is similar to the di-nucleotide compositions with skips of 1, 2, 3, 4 and 5 nucleotides respectively [21]. For any nucleotide sequence of length *N*, each *g*-spaced feature set results in 16 descriptors. The frequency of the di-nucleotide *s* and *t* with *g*-gap (*g*-spaced feature value) is given by *D*_*g*_(*s*, *t*)/(*N* − 1), *where s*, *t* = *A*, *T*, *G*, *C*; *g* = 1, 2, 3, 4, 5 and *D*_*g*_(*s*, *t*) represents the counts of di-nucleotide *s* and *t* with *g*-gap. An example of computing different *g*-spaced descriptors for the di-nucleotide AA is shown in Fig. 1c. The *g*-spaced base pair features were computed by using *BioSeqClass* R-package [28], where the function *featureCKSAAP* was executed to generate the features.

### Supervised learning technique

Supervised learning methods are promising for DNA barcode-based species identification [15]. For instance, supervised learning techniques namely SVM (with sequential minimal optimization) [29], C4.5 (J48) [30], RIPPER [31] and Naïve Bayes [32] were employed by Weitschek et al. [15] for species identification based on DNA barcode. In SPIDBAR [26], RF supervised learning technique was applied for prediction of species using barcode sequences. Specific to the fungal species identification, Naïve Bayes algorithm was employed in RDP [16], SINTAX [1] and Mycofier [18], whereas *k*NN was used in MOTHUR [19]. Motivated by the successful application of machine learning techniques in earlier studies, we preferred to use RF supervised learning model for identification of fungal species in the present study. Here, the class labels are the species names of fungi and the number of classes is same as the number of distinct species present in the dataset. Also, there are other advantages of using RF i.e., it is non-parametric (independent of the probability distribution of the dataset), robust to noise and can handle large datasets [27]. Since there were more than two species of fungus, a multiclass RF [33] model was built for prediction of species.

### Random Forest (RF)

RF [22] is an ensemble learning method, consisting of several classification trees [34], where each classifier (classification tree) is constructed on a bootstrap resample of the learning dataset. Since each classifier is built upon a bootstrap sample, on an average 36.8% of observations do not play any role in the construction of each classification tree and are called Out-Of-Bag (OOB) instances [35]. In other words, each classifier in RF is built on 2/3^rd^ of the learning data and tested on the 1/3^rd^ OOB sample. These OOB samples are the source of data for measuring the prediction error of RF. More clearly, the error for each classifier in RF is measured based on its OOB samples (called as OOB error) and these OOB errors are averaged over all the decision trees to compute the OOB error of the forest. As far as prediction of test instance is concerned, each classifier of RF votes each test instances to one of the pre-defined *K* classes and the test instance is predicted by the label of winning class [35]. There are two important parameters in RF i.e., *mtry* (number of variables to choose at each node for splitting) and *ntree* (number of decision trees to construct in the forest), tuning of which is required to achieve maximum prediction accuracy. For tuning of *ntree*, the RF was trained by using the feature set *g*=1, *g*=1+2, *g*=1+2+3, *g*=1+2+3+4 and *g*=1+2+3+4+5 with varying number of decision tress (1 to 500) and default *mtry* ($\sqrt{\mathrm{no}.\mathrm{of}\ \mathrm{variables}}=\sqrt{\mathrm{p}}$). The number of trees after which the OOB-error rate got stabilized was considered as the optimal *ntree*. With the optimum *ntree*, RF was again trained with the same datasets with varying *mtry* values (1, $\frac{\sqrt{\mathrm{p}}}{2},\sqrt{\mathrm{p}},2\sqrt{\mathrm{p}},3\sqrt{\mathrm{p}},\frac{\mathrm{p}}{2},\mathrm{p}$). The *mtry* that generated the lowest OOB-error rate was considered as the optimal *mtry*. A flow chart describing the process involved in prediction using RF method is shown in Fig. 1d. For implementing RF methodology, the function *randomForest* available in the R-package “randomForest” [36] was used.

### Training and validation

At least four sequences per species (class) are required to train the supervised learning classifier for species identification using DNA barcode [15]. However, we have considered those species for which at least three sequences were also available. Here, seven different datasets were prepared with 3, 4, 5, 6, 7, 8 and 9 sequences per species respectively. The sequences in these datasets were randomly drawn from the original dataset. Number of sequences and species for each category are given in Table 2. For the dataset with *k* sequences per species, a *k*-fold CV procedure [37] was employed to evaluate the species identification success rate (SISR) of the proposed model. For *k*-fold CV, *k* subsets were prepared by randomly splitting the whole dataset in such a manner that one sequence of each species was present in each subset. In the *k*-fold CV procedure, *k-1* subsets were utilized for training of the model and the rest one subset was utilized for validating the corresponding trained model in each fold. In this procedure, all the *k* subsets were provided equal opportunity to be used as validation set, where the accuracy was measured in terms of SISR averaged over *k* folds of the CV. The SISR is defined as follows:

**Table 2:** Number of sequences, species, sequences/species for the considered seven categories of datasets. For instance, in the first category there are 3770 species with 11210 sequences, where each species has 3 sequences. Further, in the category with *k* sequences per species, a *k*-fold cross validation was adopted where *k*-1 sequences per species were used to train the model and rest one sequence was used to assess the model accuracy.

#Sequence/Species	3	4	5	6	7	8	9
#Species	3770	3461	2777	2328	1998	1773	1498
#Sequence	11210	13844	13885	13968	13986	14184	13482

Let *N*_*h*_ be the number of query sequences belong to the *h*^*th*^ species (class) and *n*_*h*_ be the number of query instances correctly classified into *h*^*th*^ class, where *h=1, 2, …, H*. Then the SISR can be computed as$\sum \limits_{h=1}^H{n}_h/\sum \limits_{h=1}^H{N}_h$.

### Prediction for other species

To check the suitability of the proposed approach for the prediction of other species (other than fungi), its performance was assessed on five different taxonomical entities namely *Inga*, *Drosophila, Cypraiedae,* Fish and Bat. The barcode sequences for these entities were retrieved from http://dmb.iasi.cnr.it/blog.php, which have also been utilized in earlier developed species identification methods [15, 38]. The numbers of sequences for the reference and query datasets for these entities are given in Table 3.

**Table 3:** Summary of the training and test datasets for five different taxonomical entities.

Dataset	Taxonomical entity				
	*Drosophila*	*Inga*	Fish	Bat	*Cypraiedae*
#Train (reference)	419	791	515	682	1656
#Test (query)	116	122	111	144	352

#Train: Number of sequences in the training set

#Test: Number of sequences in the test set

### Prediction with simulated datasets

To assess the robustness of the proposed model, its performance was also evaluated using simulated datasets that were generated by Weitschek et al. [15]. There were three datasets with effective population sizes (*Ne*) 1000, 10000 and 50000, where 100 sets were present in each dataset and the sequences in each set were belonged to 50 species. These datasets can be accessed at http://dmb.iasi.cnr.it/blog.php.

### Comparison with existing approaches for prediction of species other than fungi

The SISR of the proposed model was also evaluated against the existing similarity, tree and diagnostic- based [15] methods, for species identification other than fungi. In tree-based approaches, the labels of an unknown species are decided based on the cluster membership of their barcode sequences with that of reference dataset, where the clusters are formed by Parsimony (PAR) [39] or Neighbor joining (NJ) [40] method. The similarity-based approach assigns an unknown specimen to that species of reference library with the barcode of which maximum number of nucleotides of query barcode match, where the nucleotide matches are measured by using nearest neighbor (NN) [41] or BLAST [42] technique. Diagnostic-based methods namely DNA-BAR [43], BLOG [44] assign species label to an unknown specimen depending upon the presence/absence of certain nucleotides in DNA barcode, without relying on all the characters [40]. The comparison was made by using a diverged dataset consisting of barcode sequences of *Inga* from Plantae, *Cypraeidae* from Mollusca and *Drosophil*a from Arthropoda kingdom, which were retrieved from http://dmb.iasi.cnr.it/blog.php. The sequences of *Inga*, *Cypraeidae* and *Drosophil*a also belonged to *COI, trnTD* and *ITS* genomic regions respectively. The collected dataset contains 1654, 497 & 736 sequences in the reference set, and 354, 118 & 172 sequences in the query set for *Cypraeidae, Drosophil*a and *Inga* respectively.

### Comparison with existing fungal taxonomy prediction method

The proposed computational model was further compared with the existing fungal species identification methods namely RDP classifier, SINTAX and MOTHUR. We used the executable code of the MOTHUR (https://github.com/mothur/mothur/releases/tag/v1.40.5), RDPclassifier (https://sourceforge.net/ projects/rdp-classifier/) and SINTAX (http://www.drive5.com/ usearch/manual/cmd_sintax.html) for implementing the corresponding algorithms in our fungal datasets. The performances of the methods were evaluated with a dataset of 1363 species (10 sequences per species). Accuracies were computed over 10-fold CV, where one sequence of each species was present in each fold. We preferred to use 10 sequences per species, because the datasets upto 9 sequences/species were utilized for assessing the SISR of the proposed computational model (see subsection *Training and validation*).

## Results

### Parameter optimization analysis

**Fig. 2:**
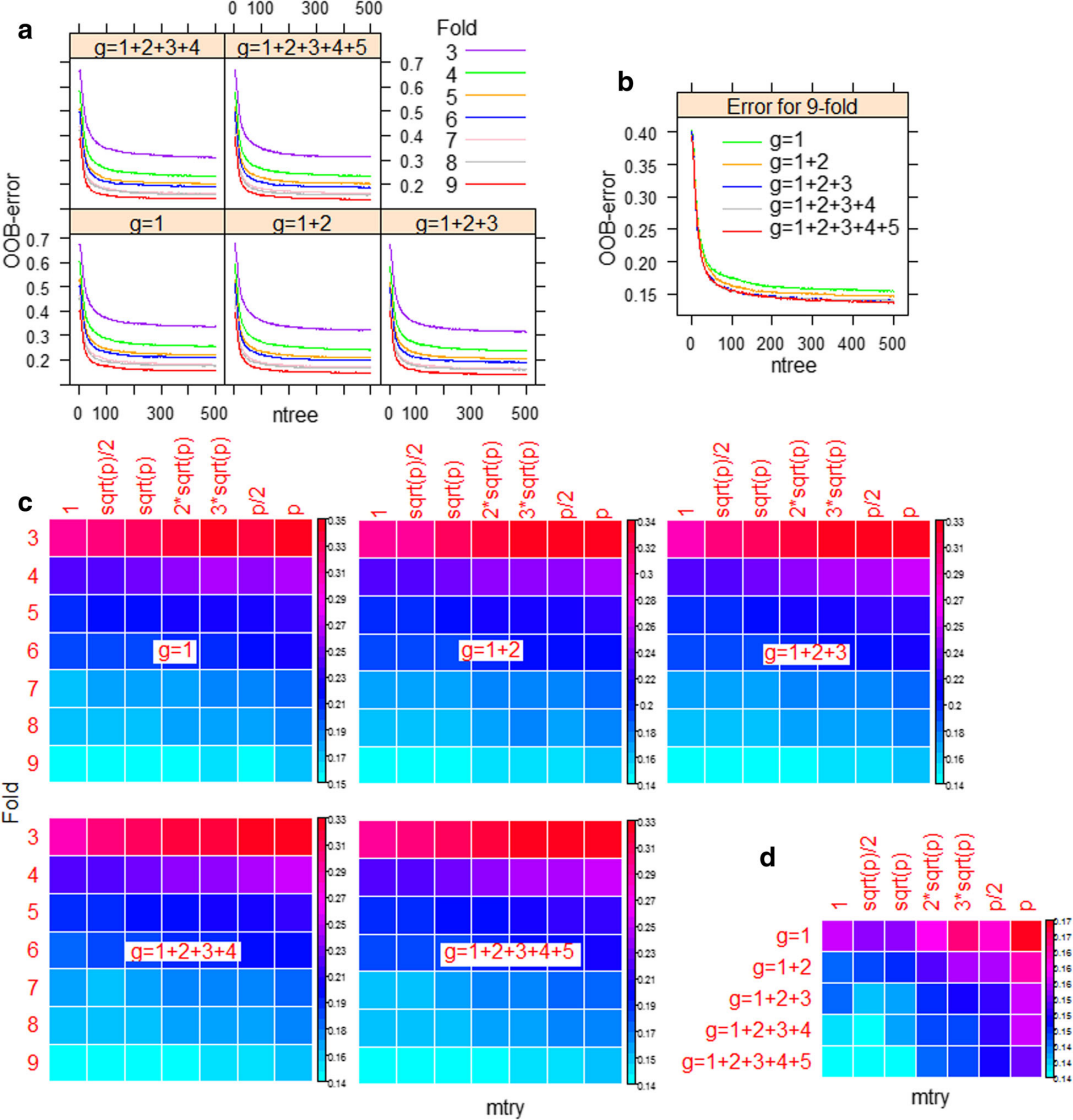
(**a**) Line graphs showing the trend of OOB-error rates with respect to different number of classification trees (*ntree*) in RF. **b** The OOB-error rates for different model representations with default values of *mtry* at *ntree*=500. **c** Heat maps of the OOB-error rates at *ntree*=500 with different values of *mtry* for different model representations. **d** Heat map of the OOB-error rate for the dataset with 9 sequences per species for different *mtry* values and model representations. It can be seen that the OOB-error got stabilized after reaching 400 classification trees, whereas *mtry*=$\sqrt{\mathrm{p}}\ \Big($9) was observed optimum due to less OOB-error rates as compared to the other values of *mtry*

In all the five model representations (*g*=1, *g*=1+2, *g*=1+2+3, *g*=1+2+3+4 and *g*=1+2+3+4+5) the OOB-error rates are seen to be stabilized after *ntree*=400 (Fig. 2a), for all the seven datasets (3-9 fold). It can also be seen that the OOB-errors are lower for the dataset with larger number of sequences per species. For instance, OOB-error rates are lower for all the model representation with 9 sequences per species than that of others (Fig. 2a). It is further observed that the OOB-error is lowest for *g*=1+2+3+4+5, as compared to the other model representation (Fig. 2b). Though, the errors are getting stabilized around *ntree*=400 (Fig. 2a), the optimum value of *ntree* was kept as 500 anticipating further improvement. With the optimum value of *ntree* (=500), it is further observed that OOB-errors are minimum for the dataset with 9 sequences per species for all the seven *mtry* values and five model representations (Fig. 2c). Further among the five model representations, OOB-error is seen to be lowest for *g*=1+2+3+4+5 and that is with *mtry*=$\sqrt{\mathrm{p}}$, which is the default *mtry* value (9 in the present study) in RF (Fig. 2d). Thus *g*=1+2+3+4+5 is the best model representation with lowest OOB-error, and the optimum values of RF parameters *ntree* and *mtry* are 500 and 9 respectively.

### Analysis of g-spaced base pair features

**Fig. 3:**
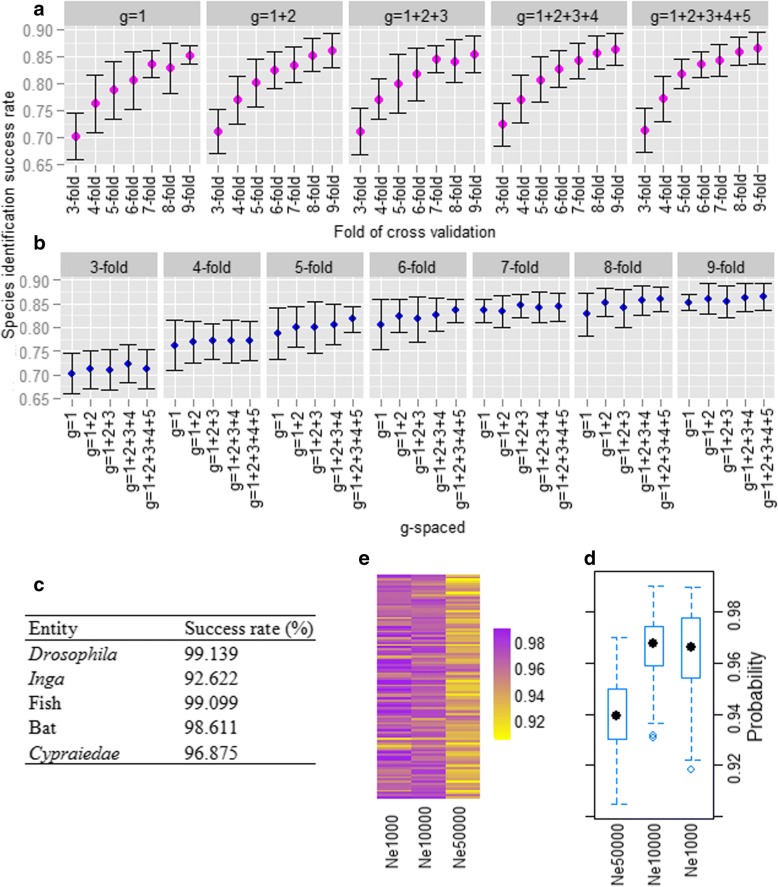
(**a**) The species identification success rates (SISR) for different combinations of *g*-spaced base pair features. **b** The SISR for different number of sequences per species. **c** The SISR of the proposed model for taxonomy prediction in *Drosophila*, *Inga*, Fish, Bat and *Cypraiedae*. **d** Box plots of the proportion of correctly predicted sequences in 100 sets of each simulated dataset. **(e)** Heat map of the proportion of correctly predicted sequence of 100 sets of each simulated dataset

Although the OOB error rate is found to be lowest for the model representation *g*=1+2+3+4+5, cross validation analysis was performed in all the five model representations (feature sets) to have a comprehensive comparative analysis. The SISRs for different number of sequences per species and for different combinations of *g* (i.e., model representations) are shown in Fig. 3. The SISRs are observed to be gradually increased while numbers of sequences per species are increased, for all the combinations of *g* (Fig. 3v). In particular, SISR reached 80%, when 4 sequences per species are used to train the model (Fig. 3a). The success rates are observed to be higher for *g*=1+2+3+4+5 as compared to *g*=1, *g*=1+2, *g*=1+2+3 and *g*=1+2+3+4. Also, it is seen that the SISRs are ≥80% for all the model representations, when ≥5 sequences per species are used for training (Fig. 3b). Though ≥80% success is achieved even for 4 sequences per species in the training dataset, that is only for *g=* 1+2+3+4 and 1+2+3+4+5. Further, SISRs are increased upto 7 sequences per species in the training set, and almost stabilized thereafter (Fig. 3a). The success rates are also found to be more stable, when the prediction model is trained with a large number of sequences (Fig. 3a). The SISRs are further observed to be more stable, when more combinations of *g*-spaced base-pair features are used in the prediction model (Fig. 3b).

### Performance analysis based on k-mer features

In one of our recent studies [26], RF classifier along with *k*-mer feature was found performing better than the existing machine learning and rule-based approaches [15] for species identification, other than fungi. Thus, we compared the performance between *g*-spaced and *k*-mer features. Four different compositions of *k*-mer (*k*=1, 2, 3 and 4) features were employed here for fungal species identification using RF classifier. Since SISR reached ~80% when 4 sequences per species were used to train the prediction model (Fig. 3a), the datasets with 5 and 6 sequences per species were only used to compare the SISR of *k*-mer feature vector with that of model representation *g=*1+2+3+4+5. The results of the comparison in terms of SISRs are given in Table 4. The SISRs are observed to be higher for larger combinations of *k*-mer features. At the same time, the accuracies were also found to be more stable both for *k*-mer and *g*-spaced features, when large number of sequence per species were included for training. Though the accuracies for *k*-mer and *g*-spaced feature sets are observed at par, the number of features for *k*-mer are larger than that of *g*-spaced feature sets. For instance, the number of features for *k*-mer 1+2+3+4 is 340 which is much larger than that of *g*=1+2+3+4+5 feature set (Table 4). Thus, it may be said that *g*-spaced features are more efficient in capturing the variability of the nucleotide distribution present in the barcode sequences of fungal species.

**Table 4:** Species identification success rates for different combinations of *k*-mer and *g*-spaced feature sets, where 4 and 5 sequences per species were used to train the prediction model. It can be seen that though the species identification success rates for both feature sets are at par, number of *k*-mer features used are larger than that of g-spaced features.

Feature-type	Feature combination	#Features	#Sequences/Species	
			5	6
*k*-mer	1+2	20	76.37±4.91	79.61±3.33
	1+2+3	84	79.21±4.71	82.72±2.81
	1+2+3+4	340	80.61±4.03	83.68±2.85
*g*-spaced	g=1+2+3+4+5	96	81.74±2.72	83.49±2.36

### Performance analysis in other species

The SISRs of the proposed approach (RF with feature set *g*=1+2+3+4+5) are shown in Fig. 3c. From the figure, it can be seen that the SISRs for other species are much higher (>92%) as compared to that of fungi (<90%). It is further observed that the SISR is low in plant (*Inga*) than that of others, and this may be due to the fact that except *Inga*, others are from animal kingdom [45]. It is further noticed that the SISRs in animal and plant species are higher than that of fungi and this may be due to the fact that in fungi ITS regions are used as barcodes which are not highly conserved as that of COI or trnTD [12]. Nevertheless, the SISRs are observed between 92-99%, and thus the proposed approach may be efficiently employed for identification of species other than fungi based on DNA barcode.

### Performance analysis using simulated datasets

With the feature set *g*=1+2+3+4+5 and RF classifier (*ntree*=500, *mtry*=9), the median of the prediction accuracies are observed to be >96% for the effective population sizes 1000 and 10000, whereas it is ~94% for 50000 (Fig. 3d). Further, the prediction accuracies are seen to be declined with increase in the effective population sizes (Fig. 3e). Nonetheless, >90% accuracy are observed in each set for all the three simulated datasets (Fig. 3e).

### Comparative analysis for prediction of species other than fungi

**Fig. 4:**
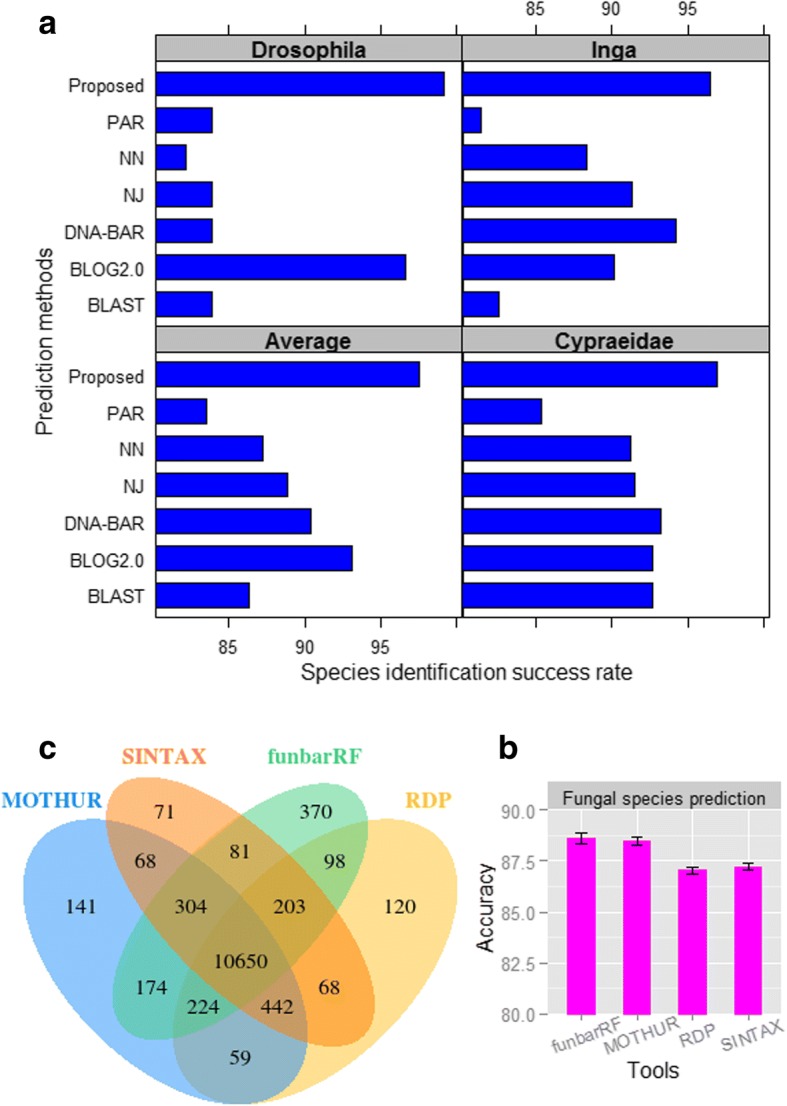
(**a**) The SISRs of the proposed model, similarity-, tree- and diagnostic-based methods for taxonomy prediction of *Drosophila*, *Inga* and *Cypraiedae.* **b** Accuracy of different taxonomy prediction method for prediction of fungal species using DNA barcode. **c** Number of correctly predicted fungal species that are common in different taxonomy prediction methods

The SISRs of the developed model (RF classifier with *g*=1+2+3+4+5 features) are ~10% higher as compared to that of similarity-based approaches (Fig. 4a). Further, diagnostic-based method outperformed the similarity- and tree-based approaches, which is corroborated with the results of Weitschek et al. [15]. Though the success rate for the diagnostic-based approach is >90%, it is ~5% less than that of proposed approach (Fig. 4a). Thus, it is inferred that the proposed approach can also achieve higher SISR than that of other ad-hoc methods for prediction of other species.

### Comparative analysis for prediction of fungal species

The accuracies of funbarRF and MOTHUR are observed ~89%, which is 2% higher than that of RDP and SINTAX algorithms (Fig. 4b). Further, the stability of the accuracy is found to be highest for RDP and lowest for funbarRF algorithm. It is also seen that 10650 correctly predicted sequences (out of 13630) are common to all the four methods (Fig. 4c). Though the SISRs are seen at par for funbarRF and MOTHUR, number of sequences predicted by funbarRF (370) that are distinct from the other classifiers are higher than that of MOTHUR (141) (Fig. 4c). This implies that the sequences that are not correctly predicted by MOTHUR are also correctly predicted by funbarRF. Thus, the funbarRF can be more efficient than MOTHUR for fungal species identification. Furthermore, ≥99% of the sequences predicted by RDP and SINTAX algorithms are found to be predicted either by MOTHUR or funbarRF or both (Fig. 4c).

### Prediction software

**Fig. 5:**
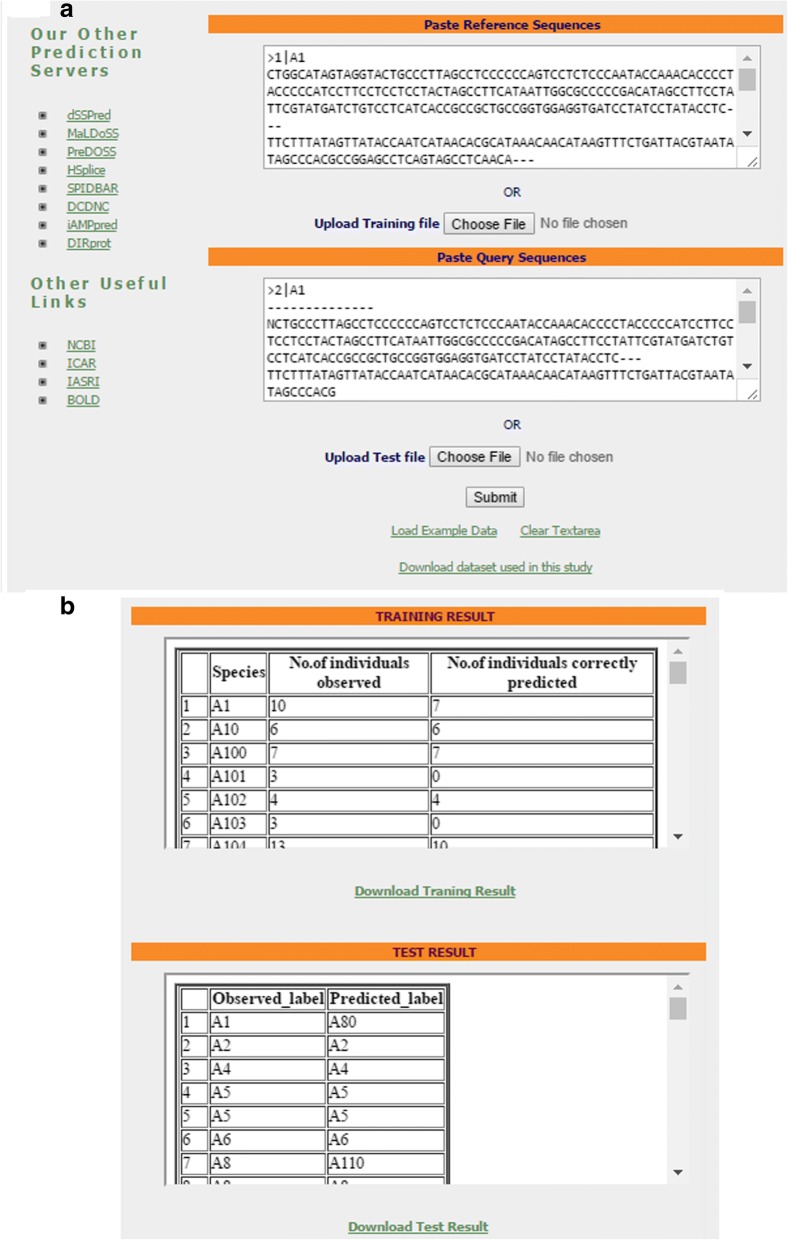
(**a**) Snapshot of the server page of the funbarRF and (**b**) result page after execution of an example dataset

Software development is an integral part, as far as the research in the field of computational biology is concerned. Here also, we have established a prediction server “funbarRF” (http://cabgrid.res.in:8080/funbarrf/) for fungal species identification. A snapshot of the server page is shown in Fig. 5a. The user interface of the server was designed using HTML, where the PHP and R-programs were implemented at the back end for execution of the proposed approach. The user has to submit both reference and query sequences in FASTA, with the sequence identifiers in BOLD format. Two result files are generated pertaining to the reference (training) and query (test) sets (Fig. 5b). Number of instances observed and correctly predicted for each reference species are given in training-result-file, whereas the predicted labels for query sequences are shown in test-result-file (Fig. 5b). To facilitate prediction using high throughput sequences, an R-package named as “funbarRF” (https://cran.r-project.org/web/packages/funbarRF/) has also been developed.

## Discussion

New species identification (taxonomically) is an integral part of biodiversity surveys that are essential for formulating policies to conserve endangered species [38]. DNA barcoding provides an alternative for molecular identification of those micro-organisms for which morphology-based species identification is often difficult [47–49]. In DNA barcoding, one of the fundamental issues is how best one can assign a correct taxonomy to an unknown specimen based on the known taxonomy of the sequences of reference library [15, 46, 50, 51]. Further, commonly used rule-based methods are dependent upon the alignment of the barcode sequences [50]. Though the alignment for coding region like COI is easier, it may not be that much easier for ITS non-coding region due to larger variability in length and indels [51]. This study presents a new computational approach that involves the feature generation based on *g*-spaced nucleotide base pairs and application of RF for identifying species using DNA barcode, with an emphasis on fungi.

The developed model was evaluated on 3770 fungal species, where the performance was analyzed based on cross validation technique. Though the identity between any two nucleotide sequences in a dataset are generally kept <80% to avoid over estimation while performing classification using machine learning techniques, this pre-processing step is mostly feasible in classification where large numbers of sequences are present in different classes. However, this pre processing step may not be feasible in the present context, because the numbers of sequences in each class (species) are very small and the numbers of classes are also larger (1498 to 3770). In other words, if such (similar) sequences are excluded, the size of the dataset will be reduced further by which the model may not be able to capture the variation present in different classes (species). We also found the similarities between sequences of different classes (species) at threshold 0.8 (results not reported), when the similarity check was performed using CD-HIT program [52]. Thus, we feel that there is a less probability of overestimation. To the best of our knowledge, we have also not found any earlier studies [15, 16, 18, 19, 26] reporting such pre-processing step, as far as species identification using DNA barcode is concerned.

Five different combinations of g-spaced base pair features were used to encode the barcode sequences that were subsequently used as input in RF classifier for species identification. Higher SISR was found for the training dataset with higher number of sequences per species. This may be due to the fact that with increase in the number of sequences per species, variability present between the species in terms of nucleotide distribution was captured more accurately.

Performances based on *g*-spaced base pair features were further compared with that of contiguous *k*-mer features, where the accuracies corresponding to 80 *g*-spaced base pair features were found similar with that of 340 *k*-mer features. This implies that, higher number of *k*-mer features may be required as compared to that of *g*-spaced features to achieve a certain level of accuracy. Though more features usually lead to a better performance, redundant features often causes misclassification and thereby reduction in classification accuracy [53]. So, one of the probable reasons for the relatively poor performance of *k*=1+2+3+4 as compared to that of *g*=1+2+3+4+5 may be that the *k*-mer features may have induced more redundancy, which may not be the case in *g*-spaced base-pair features.

We could not evaluate the proposed model on Warcup and UNITE datasets due to the constraint of computational power. However, the developed model was compared against those which were evaluated on these datasets, and found comparable accuracy for fungal species identification. Thus, the proposed model will certainly supplement the prevailing efforts for prediction of fungal species. The developed method was not compared against the Mycofier, as it has been developed for prediction of fungi at genus label. We also did not evaluate the accuracy of the developed model against PROTAX, because we found it difficult to identify the exact feature sets the PROTAX require. Moreover, the PROTAX depends upon the result of multiple sequence alignment of barcode sequences which itself takes longer time.

The developed approach was also assessed for prediction of other species. While evaluated with 5 different taxonomical entities, the proposed model achieved >90% accuracy. Besides, the proposed approach achieved >95% SISR in three diverged taxonomical entities i.e., *Drosophila*, *Inga* and *Cypraidae*, and the same was found much higher than that of rule-based approaches. Furthermore, the proposed method confirmed >90% accuracy with the simulated datasets. Therefore, it may be inferred that the developed technique is not only capable for predicting the fungal species, but also other species as well.

## Conclusion

This study presents a computational model for prediction of fungal species based on DNA barcode. The developed web server and R-package “funbarRF” will provide a platform for identification of fungi at species label. Besides, it can also be useful for identification of other species. So far so good, the proposed computational model is believed to be helpful for the taxonomists working on fungal species.

## Abbreviations

BLAST: Basic local alignment search tool
BOLD: Barcode of life data
CBOL: Consortium for barcode of life
COI: Cytochrome c oxidase subunit I
CV: Cross validation
ITS: Internal transcribed spacer
kNN: k-nearest neighbor
NJ: Neighbor joining
NN: Nearest neighbor
OOB: Out-of-bag
PAR: Parsimony
rDNA: ribosomal DNA
RF: Random forest
SISR: Species identification success rate
